# Changing from primary to secondary school highlights opportunities for school environment interventions aiming to increase physical activity and reduce sedentary behaviour: a longitudinal cohort study

**DOI:** 10.1186/s12966-015-0218-0

**Published:** 2015-05-08

**Authors:** Jennifer Marks, Lisa M Barnett, Claudia Strugnell, Steven Allender

**Affiliations:** WHO Collaborating Centre for Obesity Prevention, Deakin University, Geelong, Australia; School of Health and Social Development, Deakin University, Burwood, Australia

**Keywords:** Child, Adolescent, Environment, Behaviours, School transition, Physical activity, Sedentary, Screen, Longitudinal studies

## Abstract

**Background:**

There is little empirical evidence of the impact of transition from primary to secondary school on obesity-related risk behaviour. The purpose of this study was to examine the effect of a change of school system on physical activity (PA) and sedentary behaviour in pre-early adolescents.

**Methods:**

Fifteen schools in Victoria, Australia were recruited at random from the bottom two strata of a five level socio-economic scale. In nine schools, students in year 6 primary school transitioned to a different school for year 7 secondary school, while in six schools (combined primary-secondary), students remained in the same school environment from year 6 to year 7. Time 1 (T1) measures were collected from students (*N*=245) in year 6 (age 11-13). Time 2 (T2) data were collected from 243 (99%) of the original student cohort when in year 7.

PA and sedentary behaviour data were collected objectively (via ActiGraph accelerometer) and subjectively (via child self-report recall questionnaire). School environment data were collected via school staff survey. Change of behaviour analyses were conducted longitudinally i) for all students and ii) by change/no change of school. Mixed model regression analysis tested for behavioural interaction effects of changing/not changing school.

**Results:**

Sixty-three percent (*N*=152) changed schools from T1 to T2. Across all students we observed declines in average daily moderate to vigorous physical activity (MVPA) (−4 min) and light PA (−23 min), and increases in average daily sedentary behaviour (16 min), weekday leisure screen time (17 min) and weekday homework screen time (25 min), all *P*<0.05. Compared to students who remained in the same school environment, students who changed school reported a greater reduction in PA intensity at recess and lunch, less likelihood to cycle to/from school, greater increase in weekday (41 mins) and weekend (45 mins) leisure screen time (*P*<0.05) and greater encouragement to participate in sport. School staff surveys identified that sport participation encouragement was greater in primary and combined primary-secondary than secondary schools (*P*<0.05).

**Conclusion:**

Transitioning from primary to secondary school negatively impacts on children’s PA and sedentary behaviour, and has further compounding effects on behaviour type by changing school environments.

**Electronic supplementary material:**

The online version of this article (doi:10.1186/s12966-015-0218-0) contains supplementary material, which is available to authorized users.

## Background

Inadequate physical activity (PA) and excessive screen time in childhood and adolescence are concerning [1,2], because of associations with increased overweight/obesity and related health risks [3-7]. A minimum of 60 minutes moderate to vigorous physical activity (MVPA) per day is recommended for children and adolescents [8,9]. A 32 country study measuring the effect of age on MVPA identified declining prevalence of children meeting MVPA guidelines from late childhood to early adolescence [10]. In the US, MVPA declines on average by 38 minutes per year in children between the ages 9 and 15 years [11], with few children meeting guidelines by age 12–15 [12]. In Australian children aged 9–13 years, daily MVPA reduces by approximately 10 minutes per day per year [13].

Over the same developmental period children also record high and increasing prevalence of sedentary behaviours. More than half (56%) of US early adolescents exceed two hours of screen time [14]. A 2011 national survey of Australian 11–15 year olds revealed an average of 5–6 sedentary hours per day, including 2–3 hours recreational screen time [15]. PA [16] and sedentary [17] behavioural trends developed in childhood continue into adolescence and adulthood, with screen behaviour patterns magnified for children living in socio-economically disadvantaged environments [18], highlighting the need for early intervention.

Consistent calls are made for school-based [19] and system-level interventions [20] to promote healthy weight and related behaviour for both educational and longer term health benefits. Environmental influence on behaviour are well recognised [21] and for many children school is a critical environmental influence. Most school-based interventions target either the primary (junior or elementary) or secondary (high or senior) school setting [22], with a scarcity of evidence on the transition period from one school system to another. For some Australian students (aged 11–13), this transition from year 6 primary school to year 7 secondary school is associated with a complete change of school environment including a move to a different geographic and physical setting. For others attending a combined primary-secondary school system (e.g. Preparatory (age 5) to school year 9 [P-9] or 12 [P-12]), the transition from year 6 to year 7 does not require a change in school.

A change of school environment during childhood/adolescence is a very significant and often difficult milestone bringing multiple physical [23-25], and social changes [26], impacting upon academic performance and general health and well-being [24,27]. Despite the more obvious PA related disruptions of changing PE curriculum, school environment differences [28], and transport modes to school [29], empirically little is known about the impact of changing schools on PA and screen-based sedentary behaviour. Emerging evidence suggests that the primary to secondary school transition affects MVPA [30,31], although studies have reported contrasting findings. In the UK, a large study over this transition period found a decline in after-school MVPA and an increase in weekend MVPA [30]. In contrast, data from Belgium report that objectively measured weekday MVPA increased, whilst self-reported weekly PA decreased across the school transition [31]. Further evidence is needed to explore the contribution that a change of school has on PA, as distinguished from a general PA decline as children enter into adolescence, for informing areas to target for the promotion of PA in early adolescence.

The period of childhood to adolescence is recognised for declines in PA and increases in sedentary behaviour. Less well understood is the effect of the primary to secondary school transition, which occurs over the same time period and a priori would appear to have potential to impact on these behaviours. In this study we sought to assess: 1. PA and sedentary behaviour (including screen-time) change as pre-adolescents transition from primary to secondary school; 2. Whether students who change schools between year 6 and year 7 experience greater change in these behaviours compared to students who do not change school; and, 3. Differences between year 6 and year 7 PA and sedentary behaviour school environments. We hypothesised that PA and sedentary behaviour change will be greater in students who undergo a change of school compared to students who remain within the same school environment.

## Methods

### Design and sample

This was a longitudinal study following a cohort of students in year 6, their last year of primary school (age 11–13 years) into year 7, their first year of secondary school, with a change of school system and location as the exposure of interest. We recruited school children across different school types representing two different school transitions. The first transition describes children for whom year 6 and year 7 were conducted in separate schools, both in terms of geography and organisational structure. The second transition describes children who attend the same school for year 6 and year 7 (P-9 or P-12) at the same geographical location and within the same organisational structure. The latter school type will henceforth be referred as a P-12 school to distinguish from a discrete primary or secondary school.

Ethics clearance was obtained from the relevant university Human Research Ethics Committee and permission to approach Victorian government schools was received from the relevant school state authority. The sampling frame comprised all Victorian state government primary schools [32] stratified by a five level indexed socio-economic scale [33] then divided into two groups by school type: primary and P-12. Schools from both pools were randomly selected from the bottom two socio-economic strata and invited to participate by providing written consent. In Australia, with more discrete primary than P-12 schools, it is common for year 6 primary school students to disperse to multiple secondary schools the following year. Secondary schools were selected based on student secondary school enrolments identified at T1, and invited to participate at T2 by verbal and written invitation. Almost all secondary schools that participants transitioned to were in the same geographic and socioeconomic region as the corresponding feeder primary school. Informed consent was received from nine primary, six P-12 and 31 secondary schools. School staff (school principal and three teachers at each school) were invited to participate by completing a school PA environment survey. All grade 6 students at consenting schools were invited to participate, requiring informed written parental consent for each individual.

Parental consent was received for 40% (247/623) of invited students. At T1, 245 students participated in the final term of primary school in 2013 (Oct-Dec), two students not available to participate. T2 was conducted 5–8 months later with 243 participants from T1 (99% retention rate) in term 2 of secondary school (April-June 2014).

### Questionnaire

Student self-report behavioural questionnaires were completed at both T1 and T2 within a school class period (approximately 40 minutes), incorporating six PA, two active transport, four screen-behaviour and three school environment questions.

PA 5-point Likert scale questions were taken from the Physical Activity Questionnaire for Children (PAQ-C) [34], a validated tool for measuring PA in children and adolescents [35]. One question asked “how often were you very active” in physical education (PE) classes in the last seven days, on a scale of 1 “I don’t do PE” to 5 “always” where being very active was defined as “playing hard, running, jumping or other physical activity that makes you sweat and your heart beat faster” (excluding walking). Students were also asked what they did “most of the time” at recess and lunch on a scale ranging from 1 “sat down (talking, reading, doing schoolwork)” to 5 “ran and played hard most of the time”. The final three PA questions asked to specify from 0 up to 6 times, “how many times” in the last 7 days right after school, on school evenings, and on the last weekend “did you do sports, dance or play games in which you were very active”. These questions included an added component asking “how long did you usually spend each time? (hours/minutes)” taken from the Children’s Leisure Activities Survey (CLASS), validated as a reliable measure of PA frequency and duration in 10–12 year old children [36]. Self-report duration being very active after school and evenings were aggregated and divided by five to give average weekday for being very active outside of school hours. Self-report duration being very active on weekends was divided by two to give a daily average over the weekend.

Two active transport and three school PA environment questions were taken from the Adolescent Behaviour, Attitude, Knowledge Questionnaire (ABAKQ) [37], designed for use within an Australian school obesity prevention intervention, tested for comprehensibility and reliability [38]. Students were asked how frequent they walked/cycled to/from school (0 to 10 times per week). School environment perception questions of “how much does your school encourage all students to” 1 “play organised sport”, and 2 “be physically active at lunch time?” used a 4 point scale from 1 “a lot” to 4 “not at all”. The third question asked students to rate “the teachers at your school as role models for being physically active” on a 5-point scale from 1 “very good” to 5 “very poor”. School perception responses were reverse scored for analysis.

Screen-behaviour questions used the CLASS format [36] asking students to indicate yes/no “during a typical school day/weekend, what TV/computer activities do you usually do?” from a list comprising: watching TV, playing non-active computer games and using a computer for leisure/fun (e.g. online chatting, internet, Facebook) and homework. Students were also asked to record total hours/minutes spent on school days and weekends against each activity. Self-report duration on a typical school day and weekend watching TV, playing non-active computer games and using a computer for leisure were aggregated separately. Screen-time was capped at a maximum of 8 hours per weekday and 16 hours per two-day weekend as per CLASS guidelines [36]. Average weekday screen-time was multiplied by five, added to average weekend screen times and divided by seven to give an average daily total, then categorised as 1) meeting screen-time guidelines ≤ 2 hours/day; or 2) not meeting guidelines.

A school environment audit survey, designed to assess schools as a setting for promoting PA [38] was completed by school staff, with separate sections for school principals and teachers. School principal surveys comprised seven questions, namely the amount of time allocated to 1) recess, 2) lunch and 3) PE; the existence of 4) PA and 5) electronic devices policies; and student access to 6) indoor and 7) outdoor PA facilities using a yes/no response. School teacher surveys incorporated eleven 4–5 Likert scale PA questions, low scores representing greater adequacy/awareness/etc. and high scores represent a lower rating. Questions were as follows: 1 “What proportion of teachers are aware of the school physical activity policy?”; in the last 12 months: 2 “How good was the schools compliance with this policy?”; How adequate was the: 3 “area for outdoor play?”, 4 “area for indoor play?”, 5 “sporting and active play equipment”; 6 “How accessible was the sports equipment to all students outside of PE and sport?”; 7 “Rate the strength of the links that the school had with community sporting and recreation organisations and facilities”; 8 “What proportion of teachers at your school acted as good role models by being physically active?”; 9 “How adequate was the cycle storage facilities at your school?”; 10 “The school encouraged participation by all students in sports and other physical activities”; and 11 “How effective was your school at promoting physical activity among students?”. School PA environment responses were reverse scored for analysis.

### Accelerometry

Objective PA data was collected via the ActiGraph GT1M and GT3X+ accelerometer (ActiGraph LCC, Pensacola, US) [39,40]. The match-box sized accelerometers, worn on the right-hip, were issued to all students at T1 and 157 (64%) of participating students at T2 (due to limited accelerometer availability) to wear over a seven day period, excluding sleep and during water-based activities. T1 intra-class correlation (ICC) analyses were conducted to examine differences between students who did or did not wear an accelerometer at T2.

All accelerometers were initialised to record data at 15 second epochs on the vertical axis for consistency between models [40] and waves. Data was included for analysis using ActiLife 6 software, where minimum wear time was 480 minutes over any three 8 hour days, non-wear time defined as >60 minutes of consecutive zero counts with a 2 minute tolerance for optimal sample size and minimisation of any potential exclusion bias [41,42]. Evenson equation cut points were used to categorise accelerometer measures of metabolic equivalents (METs) on the vertical axis, as: sedentary (<1.5 METs); light (≥ 1.5 to < 4 METs); and moderate to vigorous (≥ 4 METs) [43]. Sedentary time was defined as ≤ 100 counts/min [43]. We conducted analyses on average time (minutes) spent in MVPA, light PA and sedentary time as continuous variables. We also conducted a descriptive analysis on whether recommendations of achieving at least 60 minutes MVPA per day were met, by categorising participants into two groups by meeting/not meeting MVPA guidelines of at least 60 minutes per day.

### Statistical analyses

To check for independence of schools prior to analysis, intra-class correlations were conducted by student age and sex at baseline to examine any school clustering effect. Proportions and means were calculated for student demographic variables with comparisons between students who did/did not change school. To test for changes in behaviour from T1 to T2 and differences in perceptions of school PA environments between school types, differences were assessed using exact McNemar or Bowker paired test of proportions (categorical variables), or paired *t*-test of means (continuous variables). To explore the differential effect on behaviour between changing/not changing school groups, mixed model regression analyses for longitudinal data were conducted from T1 to T2 after adjusting for accelerometer wear-time and individual scores at baseline. Separate analyses were conducted by gender. To compare school environments in relation to PA and sedentary behaviour, environmental differences were analysed using one-way ANOVA or Fishers exact test of proportions between all three school types. Comparisons between primary and secondary schools (excluding P-12 schools) were conducted using t-tests of means or Fishers exact test of proportions. All analyses were conducted using Stata 12.0 software (StataCorp LP, College Station, US). Missing data were excluded from analyses. Statistical significance was set at P < 0.05 (two-sided).

## Results

### Sample characteristics

Of 243 participants (*N* = 98 boys; 145 girls) assessed at both time points, 152 students (63%) had changed school; and 91 (37%) had remained at the same P-12 school. Mean age was 12.2 years at T1, 12.8 years at T2. The majority (85%) were born in Australia. Between change of school (CS) and no change of school (NC) groups, mean age in years (CS: 12.3 ± 0.4; NC: 12.2 ± 0.4; *NS*), sex (male) (CS: N = 58 (38%); NC: N = 40 (44%); *NS*), Australian born (CS: N = 132 (87%); NC: N = 75(82%); *NS*) and socioeconomic status (SES) (ICC 0.00; 95% CI 0–0.01) did not differ significantly at baseline. No significant school clustering effects were observed at baseline by age (ICC 0.12; 95% CI 0–0.26) or sex (ICC 0.06; 95% CI 0–0.16).

### Aim 1: PA and sedentary/screen behaviour from primary to secondary school

Minimum accelerometer wear-time criteria was achieved for 194 (80%) students at T1; 128 (82% of students who wore an available accelerometer) at T2. Analysis was conducted from 127 participant paired T1 to T2 data. No significant student differences at T1 were observed between students who did or did not meet valid accelerometer criteria at T2 by age (ICC 0.00; 95% CI 0–0.02), sex (ICC 0.04; 95% CI 0–0.18), school type (ICC 0.00; 95% CI 0–0.02) or self-report PA (ICC 0.0; 95% CI 0–0.02).

Changes in PA behaviour between primary and secondary school are shown in Table 1. Objectively measured MVPA (−4 mins/day) and light PA (−23 mins/day) declined in the six month period from T1 to T2, with minimal change in those meeting the recommended MVPA of ≥ 60 mins/day. Outside of school hours self-report ‘very active’ PA increased on weekdays (10 mins/day), but remained stable on weekends (−1 min/day; *NS*). Within a school day, 33% of students increased, whilst 15% decreased the frequency of being very active during formal physical education classes at secondary compared to primary school. Self-report PA results, revealed a significant decline in PA intensity levels at recess (*N* = 133; 55%) and lunch (*N* = 142; 60%), and decline in cycling to/from school frequency (−0.7 times/week). There were no significant differences observed in other school PA variables, namely rating teachers as PA role models, encouraging organised sport and PA lunchtime participation.

**Table 1:** **Change in physical activity from T1: Primary school to T2: Secondary school**

	**Total**	**No change**		**T1 to T2 Increase**		**T1 to T2 Decrease**		
*Categorical variable*	*N*	*N*	*%*	*N*	*%*	*N*	*%*	*p* ^†^
Average daily MVPA (accelerometer) by recommendation (1: < 60 mins; 2: ≥ 60 mins)	127	102	80%	8	6%	17	13%	
Frequency being very active in PE class (1: No PE, to 5: Always)	242	126	52%	79	33%	37	15%	*
Do most of the time at recess (1: Sit down, to 5: Run/play hard)	240	65	27%	42	18%	133	55%	*
Do most of the time at lunch (1: Sit down, to 5: Run/play hard)	236	52	22%	42	18%	142	60%	*
School encourages organised sport participation (0: Not at all, to 3: A lot)	242	109	45%	77	32%	56	23%	
School encourages physical activity at lunchtime (0: Not at all, to 3: A lot)	240	101	42%	56	23%	83	35%	
Teachers as physically active role models (1: Very poor, to 5: Very good)	242	101	42%	81	33%	60	25%	
		**T1: Primary school**		**T2: Secondary school**		**T1 to T2 Difference**		
*Continuous variable*	*N*	*Mean (SD)*	*95% CI*	*Mean (SD)*	*95% CI*	*Mean (SD)*	*95% CI*	*p* ^*#*^
Average daily (mins) MVPA (accelerometer)	127	51 (18)	49, 55	48 (17)	45, 51	−4 (13)	−6, −1	*
Average daily (mins) light PA (accelerometer)	127	219 (39)	212, 225	196 (40)	189, 203	−23 (33)	−28, −17	*
Average daily (mins) after-school being very active (self-report)	237	64 (56)	57, 71	75 (67)	66, 83	10 (66)	2, 19	*
Average daily (mins) weekend being very active (self-report)	242	84 (82)	74, 95	83 (85)	72, 94	−1 (106)	−14, 13	
Walk to/from school (0–10 times/week)	243	2.9 (3.8)	2.4, 3.4	3.0 (3.8)	2.5, 3.4	0.1 (4.1)	−0.4, 0.6	
Cycle/scoot to/from school (0–10 times/week)	243	1.2 (2.5)	0.9, 1.5	0.5 (1.6)	0.3, 0.7	−0.7 (2.6)	−1.0, −0.4	*

CI: confidence interval; MV: moderate to vigorous; PA: physical activity; PE: physical education; SD: standard deviation; * statistical significance at P < 0.05 level.

*p*†, test value for change between T1 & T2 total using exact McNemar or Bowker paired test of proportions.

*p* ^#^, test value for difference between T1 & T2 total using paired t-test of means.

The number of students who usually watched TV/videos decreased (*N* = 31; 13%) between primary and secondary school (Table 2), whereas the number of students using a computer for leisure (*N* = 49; 20%) and homework (*N* = 64; 26%) increased. Average daily duration spent using a computer for homework correspondingly increased both on weekdays (25 mins) and weekends (12 mins).

**Table 2:** **Change in sedentary/screen behaviour from T1: Primary school to T2: Secondary school**

	**Total**	**No change**		**T1 to T2 Increase**		**T1 to T2 Decrease**		
*Categorical variable*	*N*	*N*	*%*	*N*	*%*	*N*	*%*	*p* ^†^
Average daily (self-report) leisure screen time (1: ≤ 2 hrs; 2: > 2 hrs)	239	160	67%	47	20%	32	13%	
Usually watch TV/videos (0: No, 1: Yes)	241	194	80%	16	7%	31	13%	*
Usually play non-active computer games (0: No, 1: Yes)	242	173	71%	27	11%	42	17%	
Usually use computer for leisure (0: No, 1: Yes)	243	168	69%	49	20%	26	11%	*
Usually use computer for homework (0: No, 1: Yes)	243	154	63%	64	26%	25	10%	*
		**T1: Primary school**		**T2: Secondary school**		**T1 to T2 Difference**		
*Continuous variable*	*N*	*Mean (SD)*	*95% CI*	*Mean (SD)*	*95% CI*	*Mean (SD)*	*95% CI*	*p* ^*#*^
Average daily (mins) sedentary (accelerometer)	127	476 (69)	464, 488	492 (86)	477, 507	16 (76)	2, 29	*
Average daily (mins) weekday leisure screen time (self-report)^1^	240	135 (111)	120, 149	152 (114)	137, 166	17 (126)	1, 33	*
Average daily (mins) weekend leisure screen time (self-report)^1^	239	143 (121)	127, 158	158 (160)	138, 179	16 (164)	−5, 37	
Average daily (mins) weekday homework screen time (self-report)	241	36 (49)	30, 42	61 (64)	53, 69	25 (67)	16, 33	*
Average daily (mins) weekend homework screen time (self-report)	239	19 (32)	15, 24	31 (45)	26, 37	12 (48)	6, 18	*

CI: confidence interval; SD: standard deviation; * statistical significance at P < 0.05 level.

*p* ^†^, test value for change between T1 & T2 total using exact McNemar or Bowker paired test of proportions.

*p* ^#^, test value for difference between T1 & T2 total using paired t-test of means.

1. Daily screen time capped at maximum 8 hours per day as per CLASS guidelines.

Self-reported daily duration spent on screen-based activities for leisure increased on weekdays (17 mins/day) but not significantly on weekends (16 mins/day) from T1 to T2. Overall there was no significant shift in the number of students meeting the recommended maximum leisure screen time of less than 2 hours per day (*N* = 47, 20% increase; *N* = 32, 13% decrease). For the 127 participants with valid accelerometer data, total sedentary time increased from T1 to T2 of an average of 16 mins/day.

### Aim 2: Behavioural effects of changing school from T1 to T2

Mixed model regression analysis results (Table 3) identified more pronounced changes in behaviours among students who changed school compared to students who remained within the same school system from T1 to T2. The regression models show that a change of school was associated with a significant reduction in self-rated Likert scale activity intensity at recess (−1.0) and lunch (−0.9). Students who changed school, particularly girls, were 40% less likely to cycle to or from school than students who did not change school. MVPA, light PA and PE frequency were not significantly different between students who changed/did not change school.

**Table 3:** **Behavioural effects from T1 to T2 by change/no change of school and sex**

	**Change of school**		**Change to No change of school**			**Female to Male**		
**Dependent variable**	**Yes**	**No**	**Difference**		**95% CI**	**Difference**		**95% CI**
Average daily (mins) MVPA (accelerometer)^1^	−3	−4	0.8		−3.7, 5.3	−1.8		−6.5, 2.9
Average daily (mins) light PA (accelerometer)^1^	−20	−27	2.1		−8.3, 12.4	−9.5		−20.2, 1.1
Average daily (mins) after school being very active (self-report)	12	9	2.0		−15.4, 19.4	−15.3		−32.3, 1.8
Average daily (mins) weekend being very active (self-report)	−10	14	−23.2		−50.8, 4.4	24.3		−2.9, 51.4
Frequency being very active in PE class (1: No PE, to 5: Always)	0.6	0.7	0.1		−0.1, 0.4	0.0		−0.3, 0.2
Do most of the time at recess (1: Sit down, to 5: Run/play hard)	1.5	1.0	−1.0	*	−1.4, −0.6	0.2		−0.2, 0.6
Do most of the time at lunch (1: Sit down, to 5: Run/play hard)	1.5	1.2	−0.9	*	−1.2, −0.5	0.2		−0.2, 0.6
School encourages organised sport participation (0: Not at all, to 3: A lot)	0.8	0.8	0.4	*	0.1, 0.6	0.3	*	0.0, 0.5
School encourages physical activity at lunchtime (0: Not at all, to 3: A lot)	1.0	0.9	−0.1		−0.4, 0.2	−0.3	*	−0.6, −0.0
Teachers as physically active role models (1: Very poor, to 5: Very good)	0.8	0.8	0.4	*	0.2, 0.7	−0.1		−0.4, 0.1
Average daily (mins) sedentary (accelerometer)^1^	19	11	0.7		−12.3, 13.7	8.8		−4.5, 22.1
Average daily (mins) weekday leisure screen time (self-report)	32	−9	41.9	*	9.3, 74.4	24.8		−7.5, 57.1
Average daily (mins) weekend leisure screen time (self-report)	33	−13	45.9	*	3.5, 88.3	48.5	*	6.8, 90.2
Average daily (mins) weekday homework screen time (self-report)	26	23	3.5		−14.0, 21.0	0.3		−16.9, 17.5
Average daily (mins) weekend homework screen time (self-report)	14	9	4.7		−7.7, 17.2	−3.1		−15.4, 9.2
			**IRR**		**95% CI**	**IRR**		**95% CI**
Walk to/from school (0–10 times/week)	0.1	0.0	1.1		0.9, 1.2	1.2		1.0, 1.4
Cycle/scoot to/from school (0–10 times/week)	−0.7	−0.7	0.5	*	0.4, 0.8	0.6	*	0.4, 0.9
			**OR**		**95% CI**	**OR**		**95% CI**
Usually watch TV/videos	0.3	0.3	2.3		0.9, 5.9	1.0		0.4, 2.5
Usually play non-active computer games	0.4	0.3	1.2		0.7, 2.1	0.5	*	0.3, 0.9
Usually use computer for leisure	0.5	0.5	1.6		0.8, 2.8	1.2		0.6, 2.2
Usually use computer for homework	0.7	0.6	0.9		0.4, 1.9	0.6		0.3, 1.4

95% CI: 95% confidence interval; IRR: incidence rate ratio; MV: moderate to vigorous; OR: odds ratio; PA: physical activity; PE: physical education.

1. Adjusted for accelerometer wear time.

*p test value statistically significant at the P < 0.05 level.

Changing schools was also associated with an increase in leisure screen time on weekdays (42 mins/day) and weekends (46 mins/day), particularly for females (49 mins/day). Conversely, students who did not change schools reduced leisure screen time on weekdays (−9 mins/day) and weekends (−13 mins/day). Other screen based activity (number of students watching TV, playing non-active computer games, using and time spent using a computer for homework) and total sedentary time were not significantly different for students that changed school from T1 to T2.

Perceptions of the school PA environment were notably different for students who changed school. Changing school had a positive effect on encouragement of organised sport participation compared to not changing school, particularly for girls. Rating of teachers as PA role models also increased for students who changed schools from T1 to T2.

### Aim 3: Comparison of school environment by school type

School environment surveys were received from 20 (45%) school principals, representing 33% (2 of 6) P-12 type schools, 78% (7 of 9) primary, and 35% (11 of 31) secondary schools. Returned surveys from 66 (49%) school teachers represented 100% (6 of 6) P-12, 89% (8 of 9) primary and 61% (19 of 31) secondary schools.

The average amount of time allocated to recess at secondary school (23 mins) was significantly less than at primary (29 mins) or P-12 (28 mins) schools (Additional file 1: Table S1). No significant differences were found by school type from school principal response surveys for amount of time allocated to lunch or number of PE classes per week. Few schools reported having a PA policy. All secondary schools reported the existence of an electronic device policy compared to P-12 (0%) or primary schools (67%). Teacher-response surveys suggest adequacy of sport equipment was significantly greater at primary schools compared to P-12, but not secondary schools. Similarly, primary schools reported greater accessibility of sporting equipment outside of PE/sport and more adequate cycle storage facilities than P-12 or secondary schools. Both primary and P-12 type schools reported greater encouragement for all students to participate in sport and physical activities than secondary schools.

## Discussion

We found support for the hypothesis that decline in activity and increase in sedentary behaviours over the primary to secondary school transition were associated with the level of disruption in school environments. Children who experienced the more disruptive transition of moving to a new school were less active during the school day, engaged in more screen time for leisure and were less likely to engage in active transport than counterparts who remained at the same school.

This study is the first to examine the effect of the school transition on sedentary behaviour and shows that leisure screen time increased in students who changed schools between primary and secondary school and conversely declined in students who did not change school. Our overall average increase of 16–17 minutes screen-time per day, and greater average increase in students changing schools over the transition were greater than that reported in previous literature [15]. There were no significant differences between groups in accelerometer derived average daily sedentary behaviour, suggesting that other specific sedentary behaviours (e.g. sitting reading) declined proportionately in the change of school group as screen time increased. Existing literature also shows that the type of leisure screen time changes as children age, with television viewing becoming less proportionate than computer/internet [44,45]. Our results suggest that developmental changes in screen-based sedentary behaviour in early adolescence may be related to disruptions in school associated environments. Further research is needed for informing potential social-based behaviour change interventions by exploring whether a change of social environment associated with changing schools has an influence on the type of sedentary behaviour students engage in (e.g. screen-based or non-screen based).

PA duration and sedentary time deteriorate with age [12,13,46] and this study provides nuance to objective [12,46] and self-report [13] studies showing that PA decline is affected differentially by type of school transition. That the primary to secondary school transition in general is associated with a decrease in daily objective MVPA contrasts with a Belgian study [31] which reported increasing objectively measured weekday MVPA over the same transition. One possible explanation for the difference is the Belgian study followed up students two years after the transition whereas the current study follow up period was six months after changing school. It is possible that the influence on health-related behaviours is most acute immediately after the transition, and as children adjust to their new environment, negative impacts of the transition on health behaviours recedes. Confidence and opportunity to use active transport may also increase when students are older and more familiar with their new school environment. A further explanation is that observed differences may be a function of dissimilar school contexts to support children over the transition period. Low SES was a school-level inclusion criterion within the current study, potentially limiting school resources or PA opportunity, whereas school SES was unavailable in the Belgium study.

School break self-report PA intensity decreased between primary and secondary school on average for all participants in the current study, which may have contributed to overall MVPA and light PA decline. Accelerometer derived MVPA at school recess and lunch breaks have been shown to decline significantly in Australian children aged 10–12 by the last year of primary school [47]. In primary school children, lower PA intensity at recess is also associated with being of low SES [48]. With a lower PA base at primary school for children of a lower SES background, a reduction after commencing secondary school is a concern. Within the UK, longer school recess breaks are associated with greater MVPA in 9–10 year old children [49]. The current study adds new insight to this previous work by exploring the effect of length of recess time on PA in light of the transition. Time allocated to recess was significantly less at secondary compared to primary school, coinciding with a greater decline in PA intensity for students who changed schools over this transition. Lunch time durations were similar by school type, offering no explanation for declining lunch time PA intensity, suggesting that other factors encouraging PA during school breaks may be at play [50]. Previous work within primary schools demonstrates the school environment to explain 40% of recess PA intensity variability [50]. Our data intimates sporting equipment is one such factor, being more accessible at primary than secondary school.

Not all aspects of the transition are negative for PA. Despite no significant change of perception in all students transitioning to secondary school, we found students who underwent a change of school environment (particularly girls) reported an increase in encouragement to participate in organised sport and observed better PA role modelling at secondary school. Although this finding was in contrast to reported staff perceptions, student perception is particularly important to consider, as PA decline in adolescent girls is well documented [51,52].

A change of system is invariably associated with cause and effect self-correcting mechanisms to counteract the impact of change [53]. This is evident in our results comparing behaviour change between the two study groups. Whilst duration in light PA and MVPA declined and sedentary time increased across all participants, there were notable differences by type of activity between those who transitioned to a different school system between primary and secondary school, and those without a change of school. A change of seasonality (from spring/summer to autumn/winter) could provide some explanation for an overall decline in PA [54], more so for adolescent girls than boys [55], but not for between group comparisons. No significant differences in objectively measured PA and sedentary time were shown between change and no-change of school groups. In contrast, significant differences were evident between groups for self-reported PA intensity during recess and lunch breaks and leisure screen time duration. This finding is not explained by the low SES characteristics of participants, which is associated with low PA [56] as the students are from similar SES backgrounds. It is possible that organised sport participation increased within the change of school group, counteracting the effect of a decline in unstructured PA at recess and lunch, as implied by student perceptions of an increase in sport participation and greater PA teacher role modelling in their new school environment. These findings demonstrate that the school environment can moderate PA decline in early adolescence, and identifies areas to target to increase PA participation (e.g. staff training, providing sporting equipment in school breaks). Interventions to increase PA at recess have had some success at elementary/primary schools [57]; this study shows the need to intervene at secondary school breaks is also critical.

This effect of the environmental hypothesis is further strengthened by our finding that those who experienced the more disruptive transition were less likely to ride or scooter to secondary school compared to their time in primary school. Modes of transport differ between secondary and primary school aged children [58,59], a change of school often entailing a dramatic change in geographic location, physical conditions (e.g. road infrastructure) and social support, each known correlates of active transport [59,60]. Cooper et al. [61] reported differential associations of the transition depending on travel modes; MVPA increased in students using active transport in secondary compared to primary school, whereas students who changed to car travel had a reduction in MVPA [61]. De Meester et al. [31] suggest changes in PA and active transport relate to differing emphases by school type, with secondary schools emphasising PA within school hours, compared to an after-school PA focus at primary school. Others have demonstrated a variety of factors contribute to students active transport [62], though none have examined this in terms of the scale by type of primary-secondary school transition.

This longitudinal cohort study achieved a very high retention rate of cohort participants. Almost complete data minimised any potential bias from loss to follow up. Further strengths were the randomisation and independence of primary and P-12 schools at baseline. In addition, the incorporation of both objective and self-reported measures enabled analyses to be conducted by total measure and by periods and type of activity/inactivity. There is a risk of potential bias using self-reported PA measures due to individual perception of intensity and duration. This limitation was partially mitigated by the longitudinal design, with participants responding to consistency of questions at both time points. Unfortunately accelerometer data were only available for 50% of participants at T2, although there were no differences at T1 between students who had valid accelerometer data at T2 and those who didn’t, reinforcing the generalizability of the sample. It is possible that collecting PA data across two different seasons (spring/early summer and autumn/early winter) may have affected PA levels to some extent. However, our collection points were not within extreme seasons i.e. summer to winter, and little seasonal effect has been found previously with Australian adolescent males [55], so seasonality is unlikely to have had a major impact. There was a relatively low response for completed principal-based school environment audit surveys, although analyses were able to be conducted with more complete teacher-derived school environment data, providing a good indication of the differences in PA school environments for the purposes of this study.

## Conclusions

Transitioning from primary to secondary school clearly impacts on children’s PA and sedentary behaviour. Changing school environments results in a greater change in types of behaviour, showing this influential life stage is a critical target for the reduction of unhealthy behaviours. The observed reductions in PA intensity during school breaks between primary and secondary school represents a particular area for intervention. Providing more supportive environment in terms of availability and quality of equipment, role models in the form of staff and lead students, and opportunities to be active appears critical to reduce the impact of this transition. While much has been done to measure physical environments and their associations with health behaviours at different school levels, future research may seek to investigate the impact of social influence on behaviour change over the transition.

## Additional file

Additional file 1: Table S1. School PA environment by school type (Primary, Secondary, P-12 Primary-Secondary).
