# Supplementary material for: Changing from primary to secondary school highlights opportunities for school environment interventions aiming to increase physical activity and reduce sedentary behaviour: a longitudinal cohort study
Source: Int J Behav Nutr Phys Act. 2015 May 8;12:59. doi: 10.1186/s12966-015-0218-0 (PMC4436807; doi:10.1186/s12966-015-0218-0)
Supplement: Additional file 1: Table S1. — School PA environment by school type (Primary, Secondary, P-12 Primary-Secondary). [file 12966_2015_218_MOESM1_ESM.doc]

**Table S1**: School PA environment by school type (Primary, Secondary, P-12 Primary-Secondary)

|  |  |  | **School Type** | | | | | | |  |  |
| --- | --- | --- | --- | --- | --- | --- | --- | --- | --- | --- | --- |
| **Characteristic** |  | **P-12** | |  | **Primary** | |  | **Secondary** | | *p*^†^ | *p^#^* |
| ***School Principal response (2 P-12; 7 Primary; 11 Secondary Schools)*** | | ***N*** | ***Mean (SD)*** |  | ***N*** | ***Mean (SD)*** |  | ***N*** | ***Mean (SD)*** |  |  |
| Allocated time (mins) for recess |  | 2 | 28 (9) |  | 7 | 29 (4) |  | 11 | 23 (4) |  | ***** |
| Allocated time (mins) for lunch |  | 2 | 48 (3) |  | 7 | 49 (6) |  | 11 | 46 (8) |  |  |
| Year 6 physical education classes (no.) per week |  | 1 | 2 (0) |  | 7 | 2 (0) |  |  | *N/A* |  |  |
| Year 7 physical education classes (no.) per week |  | 1 | 3 (0) |  |  | *N/A* |  | 11 | 3 (1) |  |  |
|  |  | ***N*** | ***Yes %*** |  | ***N*** | ***Yes %*** |  | ***N*** | ***Yes %*** |  |  |
| Written physical activity policy |  | 1 | 0 |  | 6 | 33 |  | 11 | 18 |  |  |
| Student outdoor facilities access outside of school hours | | 2 | 50 |  | 6 | 100 |  | 11 | 55 |  |  |
| Student indoor physical activity facility access |  | 2 | 100 |  | 6 | 50 |  | 11 | 64 |  |  |
| Written personal electronic device policy |  | 1 | 0 |  | 6 | 67 |  | 11 | 100 | ***** |  |
|  |  |  |  |  |  |  |  |  |  |  |  |
| ***School Teacher response (6 P-12; 8 Primary; 19 Secondary Schools)*** | | ***N*** | ***Mean (SD)*** |  | ***N*** | ***Mean (SD)*** |  | ***N*** | ***Mean (SD)*** |  |  |
| Proportion of teachers aware of physical activity policy | 1: Very few, to 5: All | 10 | 2.9 (1.6) |  | 14 | 4.1 (1.5) |  | 30 | 3.5 (1.5) |  |  |
| PA policy compliance in last 12 months | 1: Very poor, to 5: Very good | 10 | 3.8 (1.3) |  | 13 | 4.5 (0.9) |  | 29 | 3.7 (1.2) |  |  |
| Adequacy of outdoor play area | 1: Very inadequate, to 4: Very adequate | 11 | 3.3 (1.1) |  | 16 | 3.8 (0.4) |  | 39 | 3.5 (0.6) |  |  |
| Adequacy of indoor play area | 1: Very inadequate, to 4: Very adequate | 11 | 3.4 (0.7) |  | 16 | 3.1 (0.5) |  | 39 | 2.9 (0.8) |  |  |
| Adequacy of sport/play equipment | 1: Very inadequate, to 4: Very adequate | 11 | 3.0 (0.9) |  | 16 | 3.8 (0.6) |  | 38 | 3.4 (0.8) | ***** |  |
| Equipment accessibility outside of PE/sport | 1: Very limited, to 4: Almost unlimited | 11 | 2.5 (0.8) |  | 16 | 3.5 (0.5) |  | 39 | 3.1 (0.7) | ***** | ***** |
| Strength of community sport/recreation links | 1: Very weak, to 5: Very strong | 11 | 3.8 (1.2) |  | 16 | 3.8 (0.9) |  | 39 | 3.7 (1.0) |  |  |
| Proportion of teachers as PA role model | 1: Very few, to 5: All | 11 | 3.6 (1.1) |  | 16 | 3.3(0.9) |  | 39 | 3.0 (0.8) |  |  |
| Adequacy of cycle storage facilities | 1: Very inadequate, to 4: Very adequate | 10 | 2.9 (1.4) |  | 15 | 3.6 (0.6) |  | 33 | 3.0 (0.8) |  | ***** |
| School encouraged all student sport participation | 1: Strongly disagree, to 5 Strongly agree | 11 | 4.5 (0.7) |  | 16 | 4.6 (0.6) |  | 39 | 4.0 (0.9) | ***** | ***** |
| Effectiveness of promoting PA | 1: Not effective at all, to 4: Very effective | 11 | 3.3 (0.5) |  | 16 | 3.3 (0.6) |  | 39 | 3.3 (0.7) |  |  |
|  |  |  |  |  |  |  |  |  |  |  |  |
| SD: standard deviation; PA: physical activity; PE: physical education; * p test value statistically significant at the P<0.05 level | | | | | |  |  |  |  |  |  |
| *p*†, test value for difference between all school types total using: Fishers exact test for equality of percentages or one-way ANOVA as appropriate | | | | | | | | |  |  |  |
| *p*^#^, test value for difference between Primary & Secondary schools (excluding P-12 schools) total using Fishers exact test for equality of percentages, or t-test of means as appropriate | | | | | | | | | | | |
